# Systematic analysis of inheritance pattern determination in genes that cause rare neurodevelopmental diseases

**DOI:** 10.3389/fgene.2022.990015

**Published:** 2022-09-12

**Authors:** Soojin Park, Se Song Jang, Seungbok Lee, Minsoo Kim, Hyungtai Sim, Hyeongseok Jeon, Sung Eun Hong, Jean Lee, Jeongeun Lee, Eun Young Jeon, Jeongha Lee, Cho-Rong Lee, Soo Yeon Kim, Man Jin Kim, Jihoon G. Yoon, Byung Chan Lim, Woo Joong Kim, Ki Joong Kim, Jung Min Ko, Anna Cho, Jin Sook Lee, Murim Choi, Jong-Hee Chae

**Affiliations:** ^1^ Department of Pediatrics, Seoul National University College of Medicine, Seoul National University Children’s Hospital, Seoul, South Korea; ^2^ Department of Genomic Medicine, Seoul National University Hospital, Seoul, South Korea; ^3^ Department of Biomedical Sciences, Seoul National University College of Medicine, Seoul, South Korea; ^4^ Rare Disease Center, Seoul National University Hospital, Seoul, South Korea; ^5^ Department of Pediatrics, Seoul National University Bundang Hospital, Seongnam, South Korea; ^6^ Department of Pediatrics, Seoul National University Hospital Child Cancer and Rare Disease Administration, Seoul National University Children’s Hospital, Seoul, South Korea

**Keywords:** neurodevelopmental disorder, inheritance pattern, carrier prediction, whole exome sequencing, recessive disorders

## Abstract

Despite recent advancements in our understanding of genetic etiology and its molecular and physiological consequences, it is not yet clear what genetic features determine the inheritance pattern of a disease. To address this issue, we conducted whole exome sequencing analysis to characterize genetic variants in 1,180 Korean patients with neurological symptoms. The diagnostic yield for definitive pathogenic variant findings was 50.8%, after including 33 cases (5.9%) additionally diagnosed by reanalysis. Of diagnosed patients, 33.4% carried inherited variants. At the genetic level, autosomal recessive-inherited genes were characterized by enrichments in metabolic process, muscle organization and metal ion homeostasis pathways. Transcriptome and interactome profiling analyses revealed less brain-centered expression and fewer protein-protein interactions for recessive genes. The majority of autosomal recessive genes were more tolerant of variation, and functional prediction scores of recessively-inherited variants tended to be lower than those of dominantly-inherited variants. Additionally, we were able to predict the rates of carriers for recessive variants. Our results showed that genes responsible for neurodevelopmental disorders harbor different molecular mechanisms and expression patterns according to their inheritance patterns. Also, calculated frequency rates for recessive variants could be utilized to pre-screen rare neurodevelopmental disorder carriers.

## Introduction

Genetic disorders are caused by various alterations in gene function. According to the Online Mendelian Inheritance in Men (OMIM) compendium, 4,617 genes and their variants are associated with human disease as of April 2022 (Amberger et al., 2015; Amberger and Hamosh, 2017). Broadly, disease-associated alterations can be categorized as resulting in either gain-of-function (GoF) or loss-of-function (LoF) of a gene. GoF is mostly associated with dominant inheritance, whereas LoF can appear in recessive form as well as dominant (as haploinsufficiency) (Jimenez-Sanchez et al., 2001; Schuster-Böckler and Bateman, 2008). Mendelian disorders with recessive inheritance patterns are primarily observed in ethnic groups with high rates of consanguineous marriages (Verma and Puri, 2015; Abouelhoda et al., 2016), whereas those with dominant and recessive inheritance patterns appear in comparable rates in other “outbred” ethnic groups (Baird et al., 1988). Despite recent efforts into patient genome sequencing, diagnosis, and the discovery of novel genes that cause rare Mendelian disorders, it is not yet clear what drives genes to carry variants that are inherited in dominant or recessive patterns. It seems intuitive to postulate that genes that cause diseases in recessive pattern are less critical and more tolerant in development and physiology as one has to carry defective variants in both alleles for a disease to manifest. Nevertheless, there is need to systematically evaluate whether other properties that may represent gene function, expression, and previous disease associations also play a role in the process.

The study of genes that cause recessive diseases is important because knowledge of such genes can be utilized to predict marriages between carriers and avoid the generation of new patients. This strategy has been already proven successful in a number of diseases, such as β-thalassaemia and Tay–Sachs disease (Kaback, 2001; Cao and Kan, 2013). However, those diseases are single gene disorders and display ethnic biases, making the process of variant curation and evaluation for pathogenicity and also the prediction of patients more efficient. Meanwhile, applying such an approach to diseases with heuristic symptoms requires considerably more effort because of the involvement of many genes and variants and the diverse clinical symptoms. Therefore, understanding the features of genetic variants that cause disease in a recessive inheritance pattern will provide a novel approach for avoiding generation of patients.

Complex structure and function of brain involve coordinated expression and function of many genes, and this is also reflected on a diverse array of rare Mendelian neurodevelopmental diseases (NDD) that we observe from patients. Such patients display abnormal brain function and/or structure which may affect motor function, learning ability, development, language, and other brain activities. Diagnosis of such diseases is a challenge because of the extreme genetic heterogeneity and rare occurrence, while whole exome sequencing (WES) has enhanced the yield of NDD diagnoses in clinical practice (Yang et al., 2013; Iglesias et al., 2014; Lee et al., 2014; Srivastava et al., 2014; Yang et al., 2014; Retterer et al., 2016; Trujillano et al., 2017).

Here we report analyses of the factors that confer effects on genes that cause diseases in dominant or recessive inheritance pattern. Based on a cohort of 1,180 Korean Neurodevelopmental Disorder (KND) patients and additional patient datasets from Deciphering Developmental Delay (DDD; *n* = 13,500) and the Simons Foundation Autism Research Initiative (SFARI; *n* = 34,868), we found that genes which follow a recessive inheritance pattern are more tolerant, harbor variants of lower functionality, interact with fewer other proteins, and are more enriched in metabolic and mitochondrial functional categories when compared to those that follow a dominant inheritance pattern. In addition, the ongoing accumulation of carrier information suggests possible future utility for carrier prediction as more NDD patient data become available.

## Materials and methods

### Patients and study criteria

Patients with NDD and their parents who visited the Seoul National University Children’s Hospital (SNUCH) pediatric neurology clinic were recruited to this study. Informed consent and blood samples for genomic DNA were obtained under the approval of the Seoul National University Hospital (SNUH) internal review board (#1406-081-588). Patients with confirmed genetic variants identified through candidate gene sequencing, targeted gene sequencing panel, microarray, metabolic work-up, brain/spine MRI, or muscle biopsy were excluded. A total of 1,180 patients with complex neurological symptoms of suspected genetic origin were selected by the pediatric neurologists in SNUCH.

### Whole exome sequencing

Genomic DNA was extracted from whole blood using the QIAamp DNA Blood Midi Kit according to the manufacturer’s instructions (Qiagen, Valencia, CA). WES procedures including exome capturing and sequencing were performed at Theragen Etex Bio Institute (Suwon, Korea). The data were analyzed as described previously (Lee et al., 2020). Briefly, Burrows-wheeler Aligner [v.0.7.15 (Li and Durbin 2010)] was used to align sequenced reads and the Picard software [v.2.8.0 (Broad Institute, 2019)], samtools [v.1.8 (Li et al., 2009)] and Genome Analysis Toolkit [GATK, v.4.1.4 (Mckenna et al., 2010)] were used for subsequent data processing steps such as removal of PCR duplicates, base recalibration, and variant quality control. ANNOVAR and SnpEff were used for variant annotation (Wang et al., 2010; Cingolani et al., 2012).

### Gene expression analysis

The normalized transcript level (TPM) of each gene was extracted from the Genotype-Tissue Expression (GTEx) project [v8 (GTEx Consortium, 2020)]. The median TPM value across all brain regions for each gene was divided by the median value of all other regions to determine relative brain vs. body expression. These relative TPM values were then plotted to visualize the distribution of the gene set. RNA-seq data and exon microarray data from BrainSpan (http://www.brainspan.org) were used to analyze brain spatial and temporal gene expression in the brain (Kang et al., 2011). This dataset contains expression from 16 cortical and subcortical structures along the full course of human brain development.

### Protein-protein interaction analysis

Protein correlation profiling of seven mouse tissues was used to explore tissue-specific PPI (Skinnider et al., 2021). The dataset contained more than 190,000 high-confidence PPIs identified with stable isotope labelling of tissues. We extracted for analysis those pairs that included proteins corresponding to the causal genes identified in the KND, DDD and SFARI cohorts.

### Evaluation of pathogenic variants

The pathogenicity of variants in our dataset was assessed with reference to multiple databases as follows. Normal population database such as gnomAD, ExAC, 1000 Genomes, and KOVA were used to evaluate dominant variants that were never seen as heterozygous and recessive variants that were never seen as homozygous or hemizygous when filtered by allele frequency of 0.001 in heterozygous status. *In silico* prediction scores such as CADD (Rentzsch et al., 2019), SIFT (Ng and Henikoff, 2003), and phyloP (Siepel et al., 2005) were used to gain information regarding whether variants were evolutionarily well conserved at the amino acid level. Disease databases such as OMIM (Amberger et al., 2015; Amberger and Hamosh, 2017; Amberger et al., 2019), HGMD (Stenson et al., 2003), and ClinVar (Landrum et al., 2016) were used to find causative genes with consideration of genotype-phenotype associations. Then the variants were classified based on inheritance patterns such as *de novo*, compound heterozygous, homozygous, and hemizygous by comparing to genotypes in the parents. Copy number variation (CNV) analysis through WES was carried out by comparing the mean coverage depth of each captured interval to the mean coverage depth of parental samples as described previously (Lee et al., 2020).

### Gene ontology analysis

To analyze disease associations and biological pathways that are enriched in a selected genes, a web-based analysis tool from Metascape (https://metascape.org/gp/index.html) was used. Conventional GO sources were used: biological process (BP), Cellular Component (CC) and Molecular Function (MF). Disease Gene Network (DisGeNET) was used for disease ontology. Results were collected and grouped into clusters for comparative analyses of biological process and disease association between gene groups.

### Statistical evaluation

Wilcox test was used to determine the statistical significance of the observed differences in functional scores for genes with different inheritance modes. The statistical significance of the expression level in boxplots was measured by a two-sample t-test. Statistical analyses were performed with R version 3.6.2.

## Results

### Genetic analysis of 1,180 patients with neurodevelopmental disorders

**FIGURE 1:**
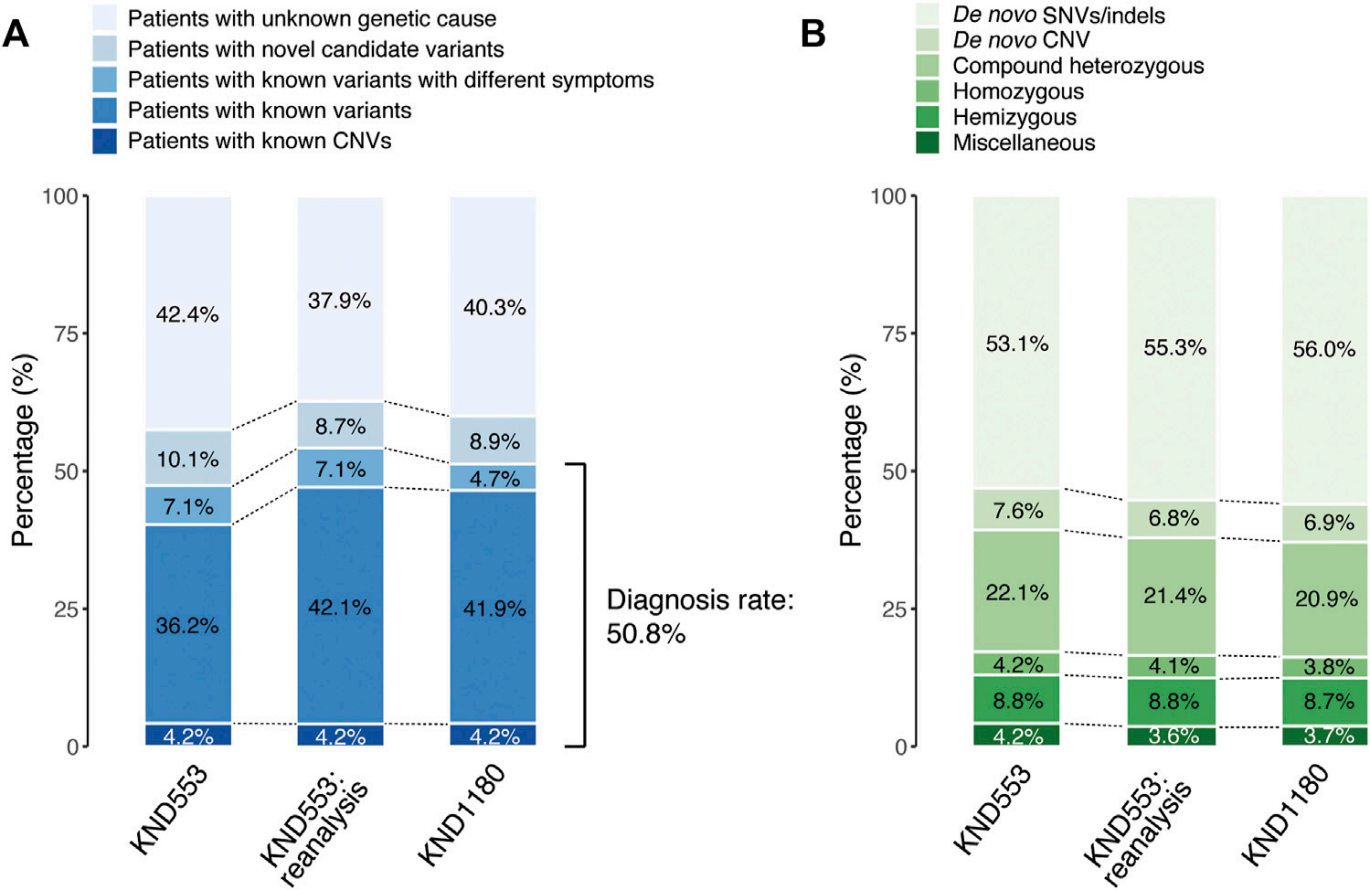
Genetic diagnosis of 1,180 KND patients (KND1180). **(A)** Diagnostic yields of the 553 KND patients in 2020 (KND553), reanalysis of KND553, and KND1180. **(B)** Breakdown of diagnosed patients by mode of inheritance.

Participants consisted of pediatric patients (mean age = 11.3 years, range 1–62) displaying one or more neurological symptoms including developmental delay, intellectual disability, intractable seizure, involuntary movements, or muscle weakness who visited SNUCH in 2014–2020 with idiopathic or undiagnosed symptoms. For genetic analysis, DNA from peripheral blood was subjected to WES. Among the 1,180 patients, parents of 707 patients were also sequenced. The resulting genome data were processed and pathogenic variants called with a standard process (Subjects and Methods). The pathogenic variants were identified in the 284 disease-causing genes in 1,180 KND patients (Supplementary Table S1). Overall, 41.9% of the patients carried known variants and 4.7% carried known variants but displayed symptoms different from those previously reported, possibly extending the disease spectra of these variants (Figure 1; Supplementary Table S2). Including the 4.2% of the patients with known CNVs (Supplementary Table S3), 50.8% of patients with pathogenic variants in known causal genes were definitively diagnosed by WES analysis (Figure 1A). This group with known variants was further divided according to variant inheritance pattern (Figure 1B). *De novo* variants were identified in more than half of diagnosed patients (62.9%) and recessive variants in about a quarter (24.7%). Lastly, 8.7% carried variants on the X chromosome with hemizygous status, making the proportion of inherited variants 33.4% (Figure 1B). This distribution of pathogenic variant inheritance is comparable to those reported in other studies using rare disease patients from outbred populations (Yang et al., 2013; Wright et al., 2015; Kuperberg et al., 2016; Marinakis et al., 2021).

### Reanalysis improved diagnostic yield

Re-evaluation of patients previously determined to be without pathogenic variants often allows for the discovery of new variants due to accumulating understanding of gene-disease relationships and improved bioinformatic pipelines (Ewans et al., 2018; Epilepsy Genetics Initiative, 2019; Jalkh et al., 2019; Fung et al., 2020). Previous re-analysis studies have reported 5%–12% increases in diagnostic yield (Ewans et al., 2018; Epilepsy Genetics Initiative, 2019; Jalkh et al., 2019; Fung et al., 2020); here, we observed a 5.9% increase in diagnostic yield, discovering pathogenic variants in 33 patients among the 553 that were previously analyzed in 2020 (KND553; Figure 1A) (Lee et al., 2020). The variants implicated in these 33 cases can be broadly divided into two groups, 1) variants for which new entries in OMIM allowed defining them as pathogenic (*n* = 8; Table 1) and 2) pathogenic calls previously missed during the bioinformatic process (*n* = 25; Table 2).

**TABLE 1:** List of newly diagnosed cases due to new gene entry into OMIM.

Index	Gene	Inheritance	Variant type	Variant	Variant status	Phenotype^a^	OMIM entry number and date of creation
1	*GEMIN5*	Rec	Comp het	c.3857A>G; *p*.Tyr1286Cys	Novel	Progressive cerebellar atrophy with severe developmental arrest	#619333; 05/19/2021
				c.2510–2A>T	Novel		
2	*STAG2*	Dom	De novo het	c.3724C>T; *p*.Arg1242*	Novel	Holoprosencephaly	#301043; 04/07/2020
3	*MED12L*	Dom	De novo het	c.1895C>T; *p*.Ser632Leu	Novel	GDD with FD	#618872; 05/02/2020
4	*DHX16*	Dom	De novo het	c.2021C>T; *p*.Thr674Met	Reported	Congenital myopathy	#618733; 01/09/2020
5	*HK1*	Dom	De novo het	c.1475C>T; *p*.Thr492Met	Reported	Severe brain atrophy, deep cortex disruption	#618547; 08/20/2019
6	*ADH5*	Rec	Comp het	c.678delA; *p*.Asp227fs	Novel	GDD, myelodysplastic syndrome	#619151; 01/13/2021
				c.832G>C; *p*.Ala278Pro	Reported		
7	*SIAH1*	Dom	De novo het	c.613G>C; *p*.Gly205Arg	Reported	GDD and FD	#619314; 05/06/2021
8	*MN1*	Dom	De novo het	c.3850delC; *p*.His1284fs	Novel	CHARGE syndrome	#618774; 02/11/2020

a GDD, global developmental delay; FD, facial dysmorphism; CHARGE, coloboma, heart defects, atresia choanae, growth retardation, genital abnormalities, and ear abnormalities.

**TABLE 2:** List of newly diagnosed cases by data re-analysis.

Index	Gene	Inheritance	Variant type	Variant	Variant status	Phenotype^b^	Reason
1	*PMM2*	Rec	Comp het	c.194A>G; *p*.Asp65Gly	Novel	Progressive cerebellar atrophy	Not clear
				c.713G>C; *p*.Arg238Pro	Reported		
2	*PDHA1*	X-linked	De novo het	c.613G>A; *p*.Val205Met	Reported	Rett syndrome-like	Not clear
3	*ZEB2*	Dom	De novo het	c.2083C>T; *p*.Arg695*	Reported	Rett syndrome-like	Not clear
4	*KAT6B*	Dom	De novo het	c.3147G>A; *p*.Pro1049Pro	Reported	Rett syndrome-like	A synonymous variant; called during re-evaluation
5	*ACTB*	Dom	De novo het	c.547C>T; *p*.Arg183Trp	Reported	Severe dystonia, ID, and SNHL	Not clear
6	*CASK*	X-linked	Hemizygous	c.1667T>G; *p*.Leu556Arg	Novel	Autism spectrum disorder with FD	Not clear
7	*MAGEL2*	Dom	De novo het	c.2873G>A; *p*.Trp958*	Reported	ID with FD and multiple anomaly	Not clear
8	*NARS2*	Rec	Comp het	c.1163C>T; *p*.Thr388Met	Novel	EE	Not clear
				c.88G>C; *p*.Val30Leu	Novel		
9	*FOXG1*	Dom	pending	c.460dupG; *p*.Glu154fs	Reported	EE and microcephaly	Not clear
10	*DYRK1A*	Dom	De novo het	c.520G>T; *p*.Val174Leu	Reported	DD with microcephaly and FD	Not clear
				c.521T>A; *p*.Val174Glu	Novel		
11	*SLC16A2*	X-linked	Hemizygous	c.1265T>G; *p*.Leu422Arg	Novel	Neurodegenerative disorder	Not clear
12	*UGDH*	Rec	Comp het	c.1183G>A; *p*.Val395Met	Novel	EE with family history	Not clear
				c.1038–2A>G	Novel		
13	*PDHA1*	X-linked	Hemizygous	c.761T>C; *p*.Leu254Ser	Reported	Leigh Syndrome	Initially missed due to coverage depth <10
14	*TBR1*	Dom	De novo het	c.1588_1594dupGGCTGCA; *p*.Thr532fs	Reported	Rett syndrome-like	Initially missed due to coverage depth <10
15	*IQSEC2*	X-linked	De novo hemi	c.2139delC; *p*.Gly714fs	Novel	Rett syndrome-like	Initially missed due to coverage depth <10
16	*SMC1A*	X-linked	Possible *de novo* het^a^	c.2923C>T; *p*.Arg975*	Reported	Rett syndrome-like	Initially missed due to coverage depth <10
17	*AHDC1*	Dom	Possible *de novo* het^a^	c.2389G>T; *p*.Glu797*	Novel	Rett syndrome-like	Initially missed due to coverage depth <10
18	*IRF2BPL*	Dom	De novo het	c.562C>T; *p*.Arg188*	Reported	Neurodegenerative disease	Called during phenotype re-evaluation
19	*UBAP1*	Dom	De novo het	c.529dupA; *p*.Met177fs	Novel	Hereditary spastic paraplegia	Called during phenotype re-evaluation
20	*GABRB3*	Dom	Possible *de novo* het^a^	c.554C>T; *p*.Thr185Ile	Reported	Rett syndrome-like	Called during phenotype re-evaluation
21	*CDK13*	Dom	Possible *de novo* het^a^	c.2149G>A; *p*.Gly717Arg	Reported	Rett syndrome-like	Called during phenotype re-evaluation
22	*TRAPPC11*	Rec	Hom	c.302A>G; *p*.Tyr101Cys	Novel	Unknown muscular dystrophy, most likely calpainopathy	Called during phenotype re-evaluation
23	*SLC35A2*	X-linked	De novo hemi	c.1A>C; *p*.Met1?	Novel	Ullrich disease or Bethlem myopathy suspected	Called during phenotype re-evaluation
24	*DHDDS*	Dom	De novo het	c.110G>A; *p*.Arg37His	Reported	EE	Called during phenotype re-evaluation
25	*KMT2C*	Dom	De novo het	c.5716C>T; *p*.Arg 1906*	Novel	Female ID, microcephaly	Called during phenotype re-evaluation

a Variant is pathogenic in ClinVar, but parental samples were not available.

b ID, intellectual disability; DD, developmental delay; SNHL, sensorineural hearing loss; FD, facial dysmorphism; EE, epileptic encephalopathy.

### Genetic characteristics of genes that follow a dominant or recessive pattern

**FIGURE 2:**
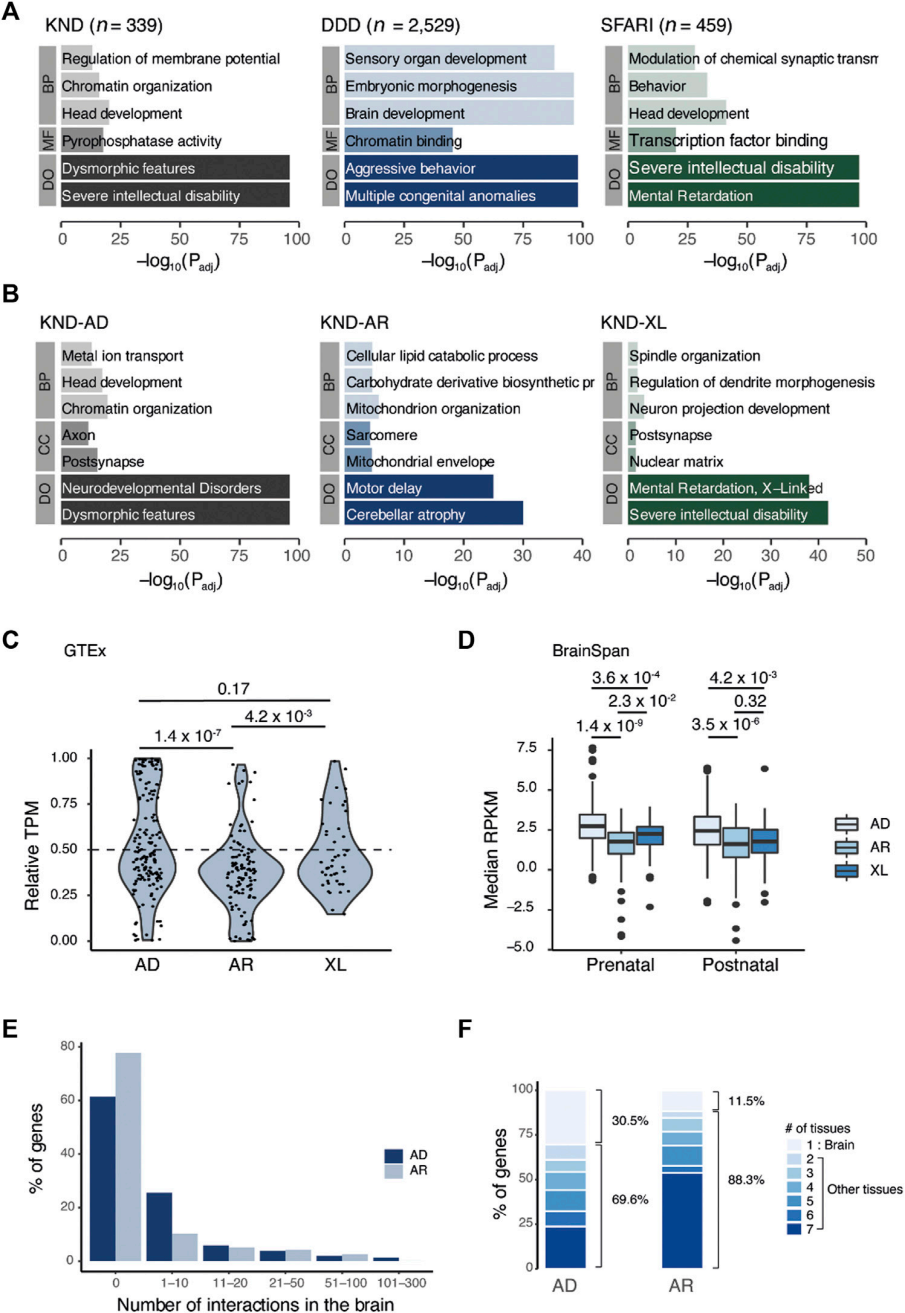
Comparative analysis of genes that cause neurodevelopmental disorders across inheritance patterns. **(A)** GO result of genes that led to the definitive diagnosis in KND, DDD, and SFARI. **(B)** Breakdown of KND genes by inheritance pattern. **(C)** Relative expression of genes in the brain vs. the body (brain/body median TPM). **(D)** Boxplot of median gene expression in the brain for two developmental periods. **(E)** Proportion of genes having the given number of PPI events in brain tissue. **(F)** Proportion of genes having the given number of PPI-positive tissues. Tissues that correspond to two to seven are heart, kidney, liver, lung, muscle, and thymus. BP, biological process; CC, cellular component; MF, molecular function; DO, disease ontology; AD, autosomal dominant; AR, autosomal recessive; XL, X-linked.

NDDs display considerable clinical and biological heterogeneity. The innate function and expression pattern of a gene can impact both its inheritance mode and the phenotype when its function or expression is altered. Understanding their genetic properties associated with inheritance modes will help in gaining a more comprehensive view of NDD. To develop this understanding, we first analyzed functional enrichments of those pathogenic genes that follow a dominant or recessive pattern in KND, and compared them with corresponding gene sets from DDD or SFARI (Figure 2A). GO analysis revealed that KND genes are enriched in molecular function and biological process terms relating to brain developmental progression, such as regulation of membrane potential, chromatin organization, head development, and pyrophosphatase activity (Figure 2A). In addition, the published variants from DDD and SFARI shared biological mechanisms involved in brain development (Supplementary Figure S1). Interestingly, differential enrichments were observed between dominant and recessive genes, with remarkably little overlap between the two gene groups (Figure 2B; Supplementary Table S4). In particular, dominant genes were strongly enriched for synaptic functions, while recessive genes were characterized by metabolic process, mitochondrial function, and muscular disease terms. Remarkably, X-linked genes shared more terms with dominant genes than with recessive genes (Figure 2B). This is unexpected because the majority of X-linked genes follow a hemizygous pattern, where disease manifests when the only X-chromosome allele in a male patient is mutated, and may follow a recessive pattern.

### Expression patterns of genes that follow dominant or recessive inheritance

Next, we used GTEx data to compare the expression profiles of KND genes in brain and other tissues to determine if expression profiles would also differ by inheritance pattern. The results showed that KND genes having brain-specific expression were more enriched in the dominant gene set compared to the recessive gene set (*p* = 1.4 × 10^−7^; Figure 2C). Interestingly, the relative brain expression of X-linked genes was more similar to dominant genes than to recessive genes. BrainSpan comprises a comprehensive survey of gene expression in the brain during development, and in the dataset, dominant genes displayed increased expression level relative to both recessive and X-linked genes (between dominant and recessive genes, *p* = 1.4 × 10^−9^ for the prenatal period and *p* = 3.5 × 10^−6^ for the postnatal period; Figure 2D). There was no substantial difference between prenatal and postnatal expression levels (Figure 2D). Therefore, our findings imply a clear distinction in function and expression level for genes of different inheritance modes.

### Tissue-specific PPI networks

PPI information enables us to explore the biological function of a protein though its physical interactions with other proteins. A recent study provided data on protein pairs that interacted in seven mouse tissues, which we used to identify PPIs for KND genes in tissue-specific context (Skinnider et al., 2021). Focusing on brain tissue, we observed that the fraction of genes with PPIs was greater among dominant genes (59/163 = 36.2%) than for recessive genes (26/117 = 22.2%), and the mean number of interactions was also higher (7.2 for dominant genes vs. 4.5 for recessive genes) (Figure 2E). Among those recessive genes having PPIs at least one interaction in the brain, more than half also interacted with other proteins in all seven tissues (14/26 = 53.8%; Figure 2F), implying these genes to have a more ubiquitous functional pattern. PPIs from DDD and SFARI were also compared for validation (Supplementary Figure S2) and yielded similar patterns of brain-specific PPIs for dominant gene products and broader PPIs for recessive gene products.

### Tolerance to pathogenic variants

**FIGURE 3:**
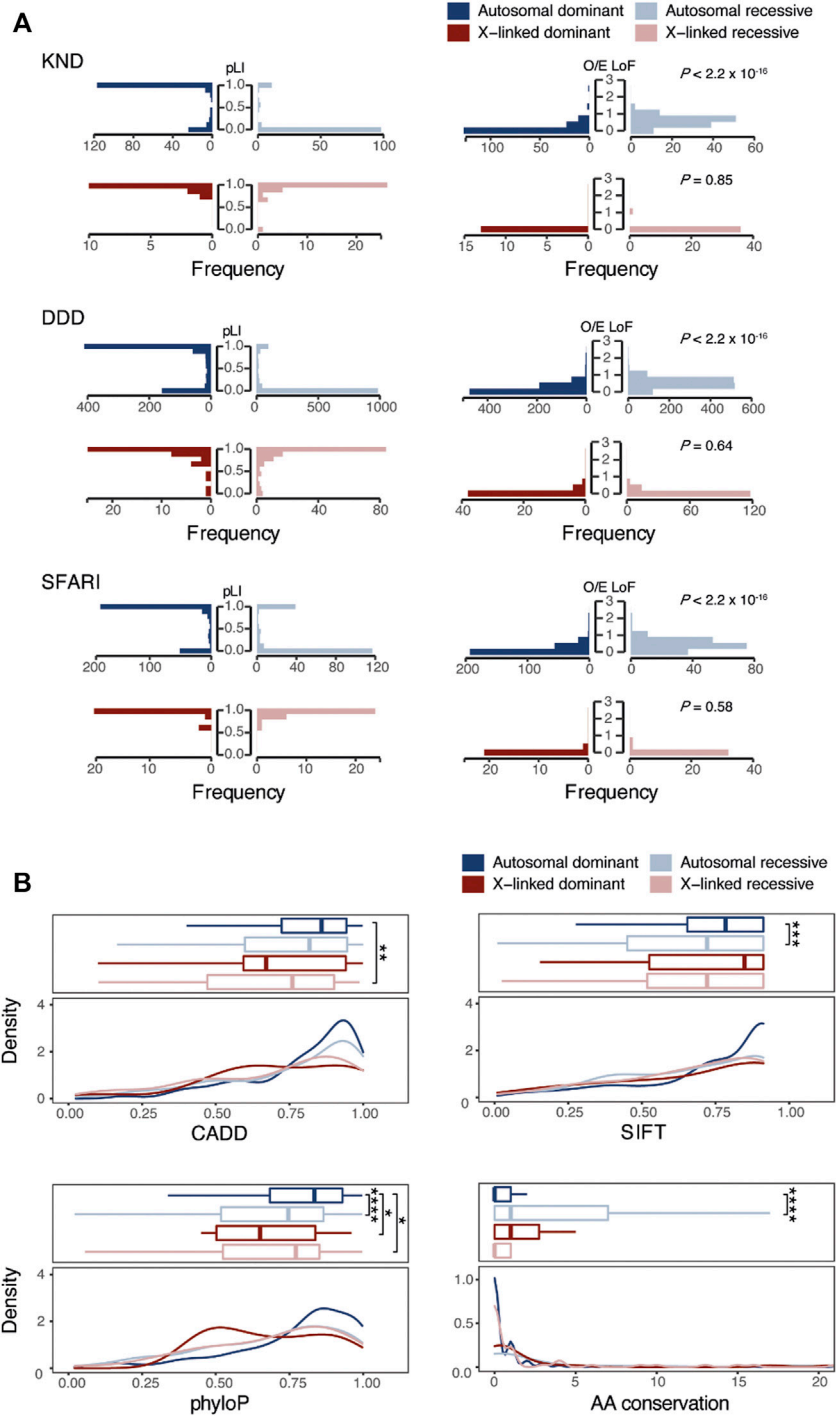
Comparison of genetic characteristics of genes and variants that follow dominant or recessive inheritance. **(A)** Comparison of pLI or O/E scores of NDD causal genes across inheritance patterns. **(B)** Comparison of functional (CADD, SIFT, and phyloP) and conservation scores among NDD causal variants according to inheritance patterns. “AA conservation” denotes the number of species with different amino acids in 99 human ortholog proteins. *, *p*-value < 0.1, **, *p*-value < 0.01, ***, *p*-value < 0.001. ****, *p*-value < 0.00001.

The tolerance of a gene, indicating the degree to which a critical mutation in it may be detrimental to human development and physiology, is effectively represented by the probability of loss of function intolerance (pLI) and the observed/expected (O/E) constraint ratio scores in gnomAD (Lek et al., 2016). In KND, autosomal dominant genes tended to have pLI values close to 1, representing strong constraint, while autosomal recessive genes showed the opposite trend with pLI close to 0 (e.g., 75.0% of autosomal dominant genes are near 1, and 84.6% of autosomal recessive genes are near 0; Figure 3A). Consistent patterns were also observed for DDD and SFARI. Meanwhile, similar to the GO analysis findings, X-linked recessive genes exhibited patterns akin to dominant genes. These observations were recapitulated when using O/E values (Figure 3A). All told, these findings suggest that genes responsible for NDDs harbor different functions according to their inheritance patterns, and they share little in terms of the molecular pathways leading to disease phenotype.

### Characteristics of variants that follow dominant or recessive inheritance

We next investigated whether the functionality of genetic variants would differ according to their inheritance pattern using functional prediction scores like CADD, SIFT, and PhyloP. Variants from DDD and SFARI were also compared for validation. This analysis revealed that functional prediction scores for recessive variants tend to be lower than those of dominant variants (between dominant and recessive variants, *p* = 0.11 for CADD, *p* = 2.2 × 10^−4^ for SIFT, *p* = 9.6 × 10^−6^ for PhyloP, and *p* = 6.6 × 10^−7^ for AA conservation; Figure 3B). This finding indicates that variants under recessive inheritance are less damaging and less critical in function, hence demonstrate little physiological effect on carriers.

### Estimating carrier frequencies of variants that cause recessive neurodevelopmental disorders

**FIGURE 4:**
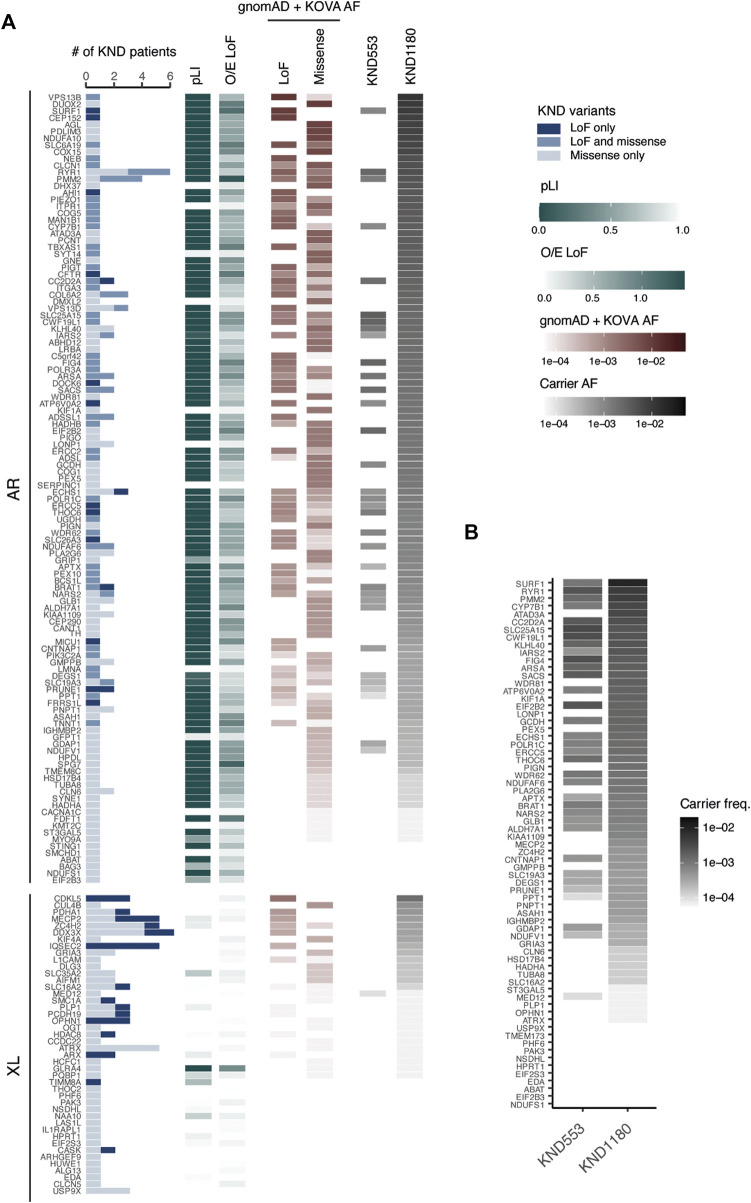
Ability to pre-determine NDD carrier status based on KND. **(A)** Heatmap displaying the number of KND patients carrying a causal variant, pathogenic variant frequency, and ability to predict carrier frequency on 161 genes. **(B)** Comparison of the ability to predict pathogenic variant carriers based on KND553 or KND1180 on the 34 overlapping genes.

In our previous study using 553 KND patients, we estimated that one in every 17 healthy individuals is a carrier for at least one pathogenic variant for a recessive genes represented in the KND cohort. This estimate remains unchanged using 1,180 patients (Lee et al., 2020), but with a patient set twice as large, we inferred that the power to predict carriers would substantially increase. We first collected a list of pathogenic LoF and missense variants from ClinVar and KND, and aggregated their population frequencies using the gnomAD East Asian and Korean Variant Archive [KOVA 2; 5,305 healthy Korean individual set (Lee et al., 2017)]. This provided us with an estimation of the number of carriers of recessive neurodevelopmental diseases in the general Korean population (Figure 4A; Supplementary Table S5). Among the 161 genes that carriers were found in KND1180 set, the estimation yields were variable by gene, ranging up to 1.2% of the general population for *VPS13B*, and no carriers were predicted for 23 genes (Figure 4A). Among the 138 genes that carriers were predicted, only 34 genes were previously found in KND553. On average, the estimation yield for KND1180 variants on the 34 overlapping genes were 1.9-fold higher than those determined for KND553 variants, implying that larger cohort size is critical for increased sensitivity (Figure 4B).

## Discussion

NDDs show considerable variability at both phenotypic and genetic levels. We conducted WES analysis to reveal genetic etiology for 1,180 undiagnosed patients in the KND cohort. Previously reported diagnostic rates of WES vary substantially among studies, ranging from 25% to 56% (Yang et al., 2013; Iglesias et al., 2014; Lee et al., 2014; Yang et al., 2014; Wright et al., 2015; Retterer et al., 2016; Jalkh et al., 2019; Marinakis et al., 2021). Herein, we report that the diagnostic yield for definitive pathogenic variant findings in KND patients was 50.8%. Among the diagnosed patients, 33.4% carried inherited variants, demonstrating that a large portion of KND patients inherited pathogenic variants from healthy parents.

It is expected that exome reanalysis applying the latest versions of databases and using improved bioinformatic tools would increase diagnostic yield (Ewans et al., 2018; Epilepsy Genetics Initiative, 2019; Jalkh et al., 2019; Fung et al., 2020). We performed reanalysis of 291 patients from the KND553 set who remained without clear pathogenic variants. This reanalysis increased diagnostic yield from 47.5% to 53.4%, which can be attributed to a number of factors: newly discovered and deposited gene-disease associations in OMIM, increased coverage allowing identification of variants that may previously have been missed, filtering out in the initial analysis of synonymous variants affecting gene splicing of *KAT6B* (*p*.Pro1049=), and re-evaluation of previously analyzed variants (Figure 1; Tables 1, 2). Therefore, we also suggest that exome sequencing data should be periodically re-evaluated.

Since NDDs may variously be caused by alterations in genes with autosomal dominant, autosomal recessive, or X-linked inheritance modes, genotype-phenotype correlations are often difficult to establish. Also, we believe that studying the fundamental differences in genes that cause NDDs in recessive or dominant mode is crucial in understanding the mechanisms of NDD pathogenicity. As a first step, we analyzed the biological pathways of the KND genes to obtain systematic insights into the molecular mechanisms associated with different inheritance modes. The results revealed that dominant and recessive genes are most strongly associated with synaptic function and metabolic processes, respectively, implying that diseases can be caused through different molecular mechanisms according to their inheritance patterns. Moreover, we observed dominant and recessive gene sets to have opposite trends in pLI and O/E scores, which proved the differences in genetic architecture between these inheritance patterns. In addition, we found gene expression profiles to also reflected this fundamental difference. Profiling of brain expression patterns in GTEx and BrainSpan revealed the dominant gene sets to exhibit specific and increased expression in the brain compared to the recessive gene set, suggesting dominant genes to be more brain-specific. In addition to the GO and expression analyses, we investigated whether PPI data support an association of tissue-specific expression and function with inheritance mode. Tissue-specific PPI networks based on direct interactions have previously demonstrated biological relevance (Skinnider et al., 2021). Here, we observed that dominant genes to have more interactions specifically in brain tissue than recessive genes. In contrast, recessive genes tended to have interactions ubiquitous across all seven tissues. Therefore, combined biological studies including PPI networks, functional pathways and phenotype data may be effective in expanding our understanding of disease progression in NDD. We also investigated variant functional effects and found that variants with recessive pathogenic alleles were less deleterious than those having dominant alleles. This is well supported by the fact that parental carriers are mostly healthy, although recent large-scale analyses have revealed heterozygous carriers of rare diseases to harbor subtle effects in various aspects of individual health and reproductivity (Barton et al., 2021; Gardner et al., 2022).

The frequency of carriers varies among population groups and specific genetic conditions could be biased toward particular ethnic groups (Rozen et al., 1999; Lynch et al., 2004; Cao and Kan, 2013; Lazarin et al., 2013). Ethnic Koreans are an outbred population, and the culture has prohibited marriages between relatives and among members of family clans for more than 500 years (Deuchler, 1992). As a major tertiary clinical institution, SNUCH covers a large portion of rare NDD patients in the country. Therefore, this study provides an unprecedented opportunity to study the occurrence of recessive diseases in an outbred population. We estimated that 24.7% of patients in the KND1180 cohort were affected by recessive conditions, which allows us to use databases such as gnomAD East Asian and KOVA to calculate carrier frequencies for reported and predicted pathogenic variants in the general population. Our resulting carrier panel will have a sensitivity of 36.1%, which is not too much deviated from previous attempts on Chinese and Israeli populations (38.7% for well-defined recessive conditions and >30% for recessive retinal diseases, respectively) (Hanany et al., 2018; Chau et al., 2022).

As expected, the larger sample size of this cohort relative to the KND553 cohort resulted in a greater number of pathogenic genes and an increase in the reported disease-associated variants enrolled in ClinVar and OMIM. Although calculated carrier frequencies may differ from those observed in clinical practices, the findings from this study will provide genetic evidence for the utility of preconception carrier screening.

## Conclusion

Recent efforts into genome-based diagnosis of rare Mendelian disorders have provided with many gene-disease relationships and understanding of disease pathophysiology. However, it has not been clearly elucidated whether there is any genetic feature that determine the modes of inheritance of sucj diseases. We took advantage of in-house as well as public patient genome data and found genetic features of recessive vs. dominant disorders. Furthermore, we demonstrate that we can utilize this understanding of recessive variants to carrier prediction to reduce future patients originated from rare recessive variants.

## Data Availability

The variant data presented in the study are deposited in the repository (https://www.sysbiolab.org/knd1180).

## Glossary

OMIM: the Online Mendelian Inheritance in Men
GoF: gain-of-function
LoF: loss-of-function
NDD: neurodevelopmental diseases
WES: whole exome sequencing
KND: Korean Neurodevelopmental Disorder
DDD: Deciphering Developmental Delay
SFARI: Simons Foundation Autism Research Initiative
SNUH: the Seoul National University Hospital
BWA: Burrows-wheeler Aligner
GATK: Genome Analysis Toolkit
TPM: Transcripts Per Million
GTEx: the Genotype-Tissue Expression
PPI: Protein-protein interaction
CNV: Copy number variation
GO: Gene ontology
pLI: the probability of loss of function intolerance
O/E: the observed/expected
KOVA: Korean Variant Archive
SNUCH: the Seoul National University Children’s Hospital
ID: intellectual disability
GDD: global developmental delay
CHARGE: coloboma, heart defects, atresia choanae, growth retardation, genital abnormalities, and ear abnormalities
FD: facial dysmorphism
EE: epileptic encephalopathy
BP: biological process
CC: cellular component
MF: molecular function
DO: disease ontology
AD: autosomal dominant
AR: autosomal recessive
XL: X-linked
