# Supplementary material for: Systematic analysis of inheritance pattern determination in genes that cause rare neurodevelopmental diseases
Source: Front Genet. 2022 Sep 12;13:990015. doi: 10.3389/fgene.2022.990015 (PMC9533195; doi:10.3389/fgene.2022.990015)
Supplement: Supplementary file 1 [file DataSheet1.docx]

Supplementary Material

## Supplementary Figures

**Supplementary figure 1.** Comparison of gene ontologies that are differently inherited in DDD and SFARI.

**Supplementary figure 2.** PPI analysis on DDD and SFARI, demonstrating more brain-specific pattern for dominant genes and more ubiquitous pattern for recessive genes.
